# Association of immune checkpoint inhibitor with survival in patients with cancers with protein tyrosine phosphatase receptor T mutation

**DOI:** 10.1002/ctm2.214

**Published:** 2020-10-27

**Authors:** Zifan He, Anlin Li, Dagui Lin, Yang Gu, Yongjian Chen, Qiyun Ou, Liren Li, Herui Yao, Yunfang Yu

**Affiliations:** ^1^ Guangdong Provincial Key Laboratory of Malignant Tumor Epigenetics and Gene Regulation Department of Medical Oncology Phase I Clinical Trial Centre Sun Yat‐sen Memorial Hospital Sun Yat‐sen University Guangzhou China; ^2^ The First Clinical Medical College Guangdong Medical University Zhanjiang China; ^3^ Department of Colorectal Surgery State Key Laboratory of Oncology in South China Collaborative Innovation Center for Cancer Medicine Sun Yat‐sen University Cancer Center Guangzhou China; ^4^ Department of Medical Oncology The Third Affiliated Hospital of Sun Yat‐sen University Guangzhou China

Dear Editor,

We and others have shown that the tumor mutation burden (TMB) and several underlying oncogenic alterations could provide clinically predictive implications for immune checkpoint inhibitor (ICI).^1^, ^2^, ^3^ Protein tyrosine phosphatases (PTPs) consist of a variety classes, and most of them are highly mutated in multiple cancers and are closely interact with innate and acquired immunity regulating immune cell activation and differentiation.^4^, ^5^ PTP receptor T (*PTPRT*) has been found to be the most frequently mutated PTP gene in cancers and could predict poor prognosis;^4^, ^6^ however, the association of *PTPRT* mutation with clinical outcomes of ICI remains unknown. Here, we performed a comprehensive pancancer investigation to clinically validate *PTPRT* mutation as a predictive biomarker for ICI therapy.

We collected clinical and *PTPRT* mutational data quantified by whole exome sequencing of 2129 cancer patients treated with ICI and 10,814 cancer patients without receiving ICI from the cBioPortal, PubMed, and The Cancer Genome Atlas. The study protocol was approved by the ethics committee of the Sun Yat‐sen Memorial Hospital of Sun Yat‐sen University. The requirement for informed consent of study participants and the permission to use the patient data were waived because the human data were obtained from publicly available datasets. All analyses were performed according to the STROBE guideline from September 18 through October 1, 2019. Overall survival (OS) were primary outcomes, which were computed using the Kaplan‐Meier method and were assessed with the log‐rank test and the hazard ratio (HR) calculated by the Cox regression model. The TMB in *PTPRT* wild‐type versus mutant groups were compared with Wilcoxon rank‐sum tests. All analyses were performed using R (version 3.4.4) and were considered statistically significant if *P* values < .05.

Among 2129 ICI‐treated patients (250 [11.7%] *PTPRT* mutant; Figure 1A), 596 (28.0%) patients had melanoma, 510 (24.0%) patients had non‐small cell lung cancer (NSCLC), and 1023 (48.1%) patients had 12 other cancer types. Patients treated with ICI showed significantly higher TMB in *PTPRT* mutant group versus *PTPRT* wild‐type group (*P* < .001; Figure 1B). Thirty‐five (6.9%) of 510 NSCLC patients and 151 (25.3%) of 596 melanoma patients harbored *PTPRT* mutation, who analogously displayed remarkably higher TMB than patients with *PTPRT* wild‐type tumors (*P* < .001; Figure 1C and D). *PTPRT* mutations were identified in 687 (6.4%) out of 10,814 patients without receiving ICI across 33 cancer types, among which the mutation frequency was 28.4% in melanoma, 11.1% in esophagogastric adenocarcinoma, 10.9% in endometrial carcinoma, 8.6% in colorectal adenocarcinoma, and 8.0% in NSCLC (Figure 2A). Missense mutations were most commonly observed (82.6%), followed by truncating mutations (15.5%) (Figure 2B).

**FIGURE 1:**
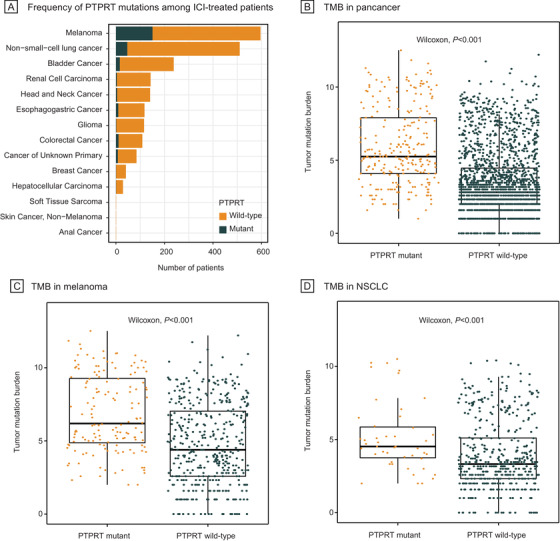
Frequency of *PTPRT* mutations and its association with tumor mutation burden during immune checkpoint inhibitor therapy. (A) Frequency of *PTPRT* mutations across 14 cancer types among patients treated with immune checkpoint inhibitor. (B‐D) Tumor mutation burden in *PTPRT* mutant versus wild‐type in pancancer, melanoma, and NSCLC, respectively. ICI, immune checkpoint inhibitor; *PTPRT*, protein tyrosine phosphatase receptor T; NSCLC, non‐small cell lung cancer

**FIGURE 2:**
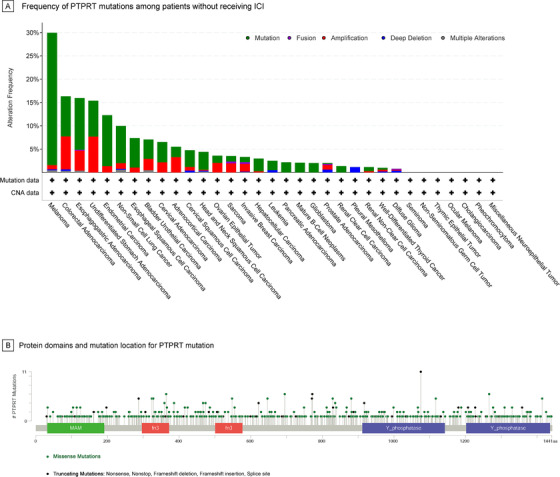
Frequency and mutation location of *PTPRT* mutations among patients without receiving immune checkpoint inhibitor. (A) Frequency of *PTPRT* mutations across 33 cancer types among patients without receiving immune checkpoint inhibitor. (B) Protein domains and mutation location for *PTPRT* mutation. Color of circle indicates corresponding mutation types. In case of different mutation types at a single position, color of the circle is determined with respect to the most frequent mutation type. ICI, immune checkpoint inhibitor; *PTPRT*, protein tyrosine phosphatase receptor T; CNA, copy number aberration; MAM, MAM domain, meprin/A5/mu; fn3, fibronectin type III domain; Y_phosphatase, protein‐tyrosine phosphatase

*PTPRT* mutation resulted in significantly longer OS in 2129 pancancer patients treated with ICI compared with *PTPRT* wild‐type (HR 0.63, 95% CI 0.52‐0.77, *P* < .001; Figure 3A). We further found the clinical usefulness of PRPRT mutation status was most prominent in ICI‐treated patients with NSCLC and melanoma. Compared with *PTPRT* wild‐type group, *PTPRT* mutation group had substantially longer OS in patients with NSCLC and melanoma (HR 0.61, 95% CI 0.48‐0.77, *P* < .001; Figure 3B). However, among ICI‐treated patients with cancers except NSCLC and melanoma, no significant difference in OS between *PTPRT* mutation and wild‐type patients was observed (HR 0.95, 95% CI 0.64‐1.43; *P *= .810).

**FIGURE 3:**
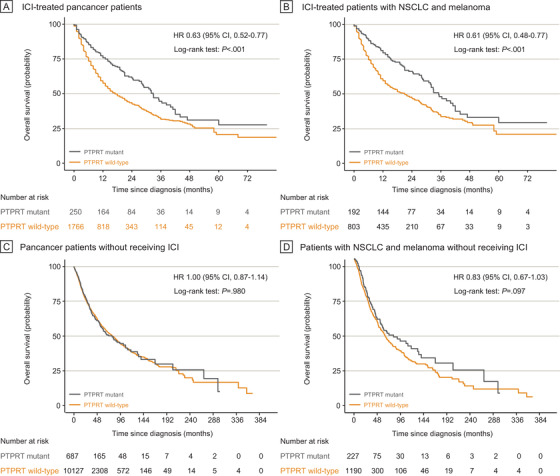
Association of *PTPRT* mutation with survival benefit of immune checkpoint inhibitor. (A) Overall survival of patients treated with immune checkpoint inhibitor in *PTPRT* mutant versus wild‐type in pancancer. (B) Same as (A) but describing patients with NSCLC and melanoma. (C) Overall survival of patient without receiving ICI in *PTPRT* mutant versus wild‐type in pancancer. (D) Same as (C) but describing patients with NSCLC and melanoma. HR, hazard ratio; CI, confidence interval; ICI, immune checkpoint inhibitor; NSCLC, non‐small cell lung cancer; *PTPRT*, protein tyrosine phosphatase receptor T

We also assessed *PTPRT* mutation in patients without receiving ICI. Among 10,814 pancancer patients, there was no difference in OS between *PTPRT* mutant and wild‐type (HR 1.00, 95% CI 0.87‐1.14; *P *= 0.980; Figure 3C). Among 986 NSCLC patients (101 [10.2%] *PTPRT* mutant) and 431 melanoma patients (126 [29.2%] *PTPRT* mutant), no difference in OS between *PTPRT* mutant and wild‐type patients was observed, either (HR 0.83 95% CI 0.67‐1.03; *P *= .097; Figure 3D). These findings indicated that the status of *PTPRT* mutation was particularly predictive of ICI treatment.

To the best of our knowledge, this is the first study to identify the mutation status of *PTPRT* as a key predictor of ICI efficacy. We found that *PTPRT* mutation conferred an elevated TMB and better survival during ICI therapy in pancancer and specifically in melanoma and NSCLC, which collaborated with our previous research^1^ showing a pronounced survival and response benefits of ICI among cancer patients with high TMB. *PTPRT* has not been suggested to be screened for mutations in current widely used gene panels such as Memorial Sloan Kettering Cancer Center's Integrated Mutation Profiling of Actionable Cancer Targets (MSK‐IMPACT) and FDA‐approved FoundationOne CDx (F1CDx). Therefore, *PTPRT* should be considered together with other known essential genes to expand the landscape of immuno‐oncological genomic panel, and should be integrated into multiomics to more fully realize the precision immunotherapy. In‐deep characterization of PTP expression pattern could be informative for understanding patterns of immune escape and the selection of candidates for immunotherapy.

Moreover, PD‐L1 inhibitor atezolizumab plus VGFR inhibitor bevacizumab plus platinum‐based chemotherapy was shown to have an encouraging survival benefit in recent randomized IMpower 150 trial.^7^ We hypothesized that the efficacy of this strategy probably further enhanced through concurrently targeting *PTPRT*, since *PTPRT* mutation was demonstrated to be promisingly predictive of immunotherapy efficacy in our study and has been found to determine bevacizumab resistance in the study conducted by Hsu et el.^8^ The study limitations included a potential random variability in the context of an exploratory analysis contributed by NSCLC and melanoma, our inability to assess the heterogeneity of other treatment between ICI and non‐ICI groups and to clarify the mechanisms underlying the interaction between *PTPRT* mutation and ICI. Future prospective trials with a larger sample size, more detailed clinical treatment information and a longer follow‐up are needed to validate the pancancer applicability of *PTPRT* mutation status and in‐deep characterize how *PTPRT* mutation interact with immune system to influence ICI benefit.

In conclusion, *PTPRT* mutation status could serve as a predictive biomarker for ICI in pancancer and specifically in NSCLC and melanoma.

## CONFLICT OF INTEREST

The authors declare that the research was conducted in the absence of any commercial or financial relationships that could be construed as a potential conflict of interest.

## ETHICS APPROVAL AND CONSENT TO PARTICIPATE

The study protocol was approved by the ethics committee of the Sun Yat‐sen Memorial Hospital of Sun Yat‐sen University. The requirement for informed consent of study participants and the permission to use the patient data were waived because the human data were obtained from publicly available datasets.
